# Assessment of thyroid function of newly diagnosed SARS-CoV-2 infected patients in Nigeria

**DOI:** 10.11604/pamj.2021.40.9.26358

**Published:** 2021-09-02

**Authors:** Chika Juliet Okwor, Ijeoma Angela Meka, Kazeem Sanjo Akinwande, Victory Fabian Edem, Vitalis Chukwuemeka Okwor

**Affiliations:** 1 Department of Chemical Pathology, University of Nigeria Teaching Hospital, Ituku/Ozalla, Enugu, Nigeria,; 2 Department of Chemical Pathology and Immunology, Federal Medical Centre, Abeokuta, Ogun State, Nigeria,; 3 Department of Immunology, University of Ibadan, Oyo State, Nigeria,; 4 Department of Radiation Medicine, University of Nigeria Teaching Hospital, Ituku/Ozalla Enugu, Nigeria

**Keywords:** COVID-19, thyroid function, thyroid stimulating hormone, thyroxine, tri-iodothyronine

## Abstract

**Introduction:**

the outbreak and rapid spread of the novel SARS-CoV-2, the causative agent of the coronavirus disease 2019 (COVID-19), has evolved into an unprecedented global pandemic. The infection impairs several human organs and systems, however, it is not clear how it affects thyroid function. The study therefore aimed at measuring plasma levels of thyroid hormones and Hs-CRP in COVID-19 patients and apparently healthy uninfected controls to assess the possible effect of SAR-CoV-2 infection on thyroid function.

**Methods:**

in this cross-sectional study carried out between May-August 2020, 90 consenting participants comprising 45 COVID-19 patients and 45 apparently healthy uninfected controls were recruited. Plasma FT3, FT4, TSH and Hs-CRP were measured using Enzyme Linked Immunosorbent Assay (ELISA) method. Data was analysed using SPSS version 20 and statistical significance set at p < 0.05.

**Results:**

the mean plasma FT3 and TSH concentrations were significantly higher in COVID-19 patients compared to controls (p < 0.001, p < 0.001 respectively). Euthyroidism was observed in all uninfected controls, whereas 35 (77.8%) COVID-19 patients were euthyroid. Sick euthyroid and subclinical hypothyroidism was observed in 7 (15.6%) and 3 (6.7%) COVID-19 patients, respectively.

**Conclusion:**

though there was a preponderance of euthyroidism among COVID-19 patients, significantly higher mean plasma levels of TSH and FT3, sick euthyroid syndrome and subclinical hypothyroidism observed among some COVID-19 patients may be indicative of disease-related thyroid function changes. Hence, there is need to pay attention to thyroid function during and after treatment of COVID-19.

## Introduction

The novel coronavirus disease 2019 (COVID-19) caused by severe acute respiratory syndrome coronavirus 2 (SARS-CoV-2) has become a pandemic and foremost public health concern ravaging the whole world, thereby constituting an immense threat to global health [1-3]. As at June 20, 2020, about 8.5 million cases had been confirmed globally with mortality of 455, 231 reported [4], which represents a case mortality rate of 5.4%. Compared to other WHO regions, the case mortality rate in Africa has been reduced, but the rising number of incident cases in this region portends an imminent threat due to weak health systems, inadequate health infrastructures and the colossal poverty status of most countries in the continent [5, 6]. As of June 20, 2020, Africa had 208, 535 confirmed cases with 4, 595 deaths, representing a case mortality rate of 2.2% [4] while Nigeria, had a total of 19, 147 confirmed cases with 487 deaths recorded thereby culminating to 2.5% case mortality rate [4].

Detrimental effects of SARS-CoV- 2 infection on multiple organs and systems of the body including respiratory, immune, digestive, circulatory, hepatic, renal and hematological systems have been reported in COVID-19 patients [7-11]. However, it is unclear if COVID-19 affects thyroid function. A detailed histopathological study on the effects of SARS-CoV infection on the thyroid gland was done during the SARS outbreak of 2002-2003 [12]. Extensive injury of the parafollicular cells and follicular epithelium with destruction and exfoliation of epithelial cells into the follicle leading to its disruption were reported. The authors attributed lower levels of thyroid hormones noted in clinical studies to these changes [12]. Previous studies also reported changes in thyroid hormone levels in SARS-CoV-2 patients during acute illness and convalescent phase [13, 14]. These studies are suggestive of a need for monitoring of thyroid hormones during clinical management of viral infections caused by the family of coronaviruses. However, in the face of the current novel SARS-CoV-2 infection, little mention or consideration is given to thyroid function in the management of infected patients.

Brancatella *et al*. [15] reported the first case of subacute thyroiditis after SARS-CoV-2 infection. Recently published pathology data of the thyroid gland from minimally invasive autopsies conducted in patients who died of SARS-CoV-2, reported no abnormalities in the thyroid follicular morphology but noted lymphocytic infiltration in the interstitium [16]. Given the mounting uncertainties surrounding the pathophysiology of the current COVID-19 disease, it is imperative to understand the status of thyroid function in COVID-19 patients by assessing the levels of thyroid hormones in these patients to ascertain the impact of SARS-CoV-2 illness on thyroid function. The present study therefore aimed at measuring plasma levels of free triiodothyronine (FT3), free thyroxine (FT4), Thyroid Stimulating Hormone (TSH) and High sensitivity C - reactive protein (Hs-CRP) in COVID-19 patients and apparently healthy uninfected controls.

## Methods

### Study design and setting

The design of the study was cross-sectional, and it was conducted at the Ogun State COVID-19 isolation center and Federal Medical Center, Abeokuta, Ogun State, Nigeria over a 5-month period (April - August 2020).

### Study population

A total of 90 consenting participants comprising 45 RT-PCR confirmed COVID-19 patients and 45 apparently healthy controls were recruited for this study. RT-PCR confirmed COVID-19 patients (SARS-CoV-2 positive) were recruited following case confirmation and admission into the Ogun State COVID-19 isolation centre. The study adopted a total population sampling technique. All patients admitted at the Ogun State COVID-19 isolation centre with confirmed SARS-CoV-2 infection from 27th April to 14th May 2020 were included in this study after obtaining written informed consent. Apparently healthy individuals were screened using the COVID-19 IgG/IgM rapid test kit (Wuhan UNscience Biotechnology Co., Ltd, China) to exclude SARS-CoV-2 infection. Age and gender matched uninfected individuals with negative COVID-19 IgG/IgM rapid test kit results, were enrolled after obtaining written informed consent. Excluded from the study were individuals previously diagnosed with thyroid dysfunction, HIV/AIDS or on any thyroid hormone replacement therapy. Pregnant women and children were also excluded from the study.

### Data collection

Relevant demographic and clinical data were collected using a researcher-administered questionnaire. These questionnaires were developed by the researchers for the purpose of the study. The first section included participants´ demographic data, while the second section included information on patients´ past medical history and current medications.

### Sample collection and Laboratory analysis

Five millilitres (5mls) of blood sample was aseptically obtained from study participants by venepuncture and dispensed into lithium heparin bottles. A trained Phlebotomist carried out blood sample collection. Samples were transported in sealed bags placed in an airtight container to the laboratory for processing. Plasma was separated by centrifugation at 4000 radians per minute, for 10 minutes in a biosafety cabinet and stored at -20°C till analysis. Ethical standards were carefully observed in the handling, storage, and disposal of research samples. Plasma FT3, FT4, TSH and Hs-CRP were measured using Enzyme Linked Immunosorbent Assay (ELISA) method according to manufacturer´s (Calbiotech Inc, USA) instructions.

### Statistical analysis

Data were entered into a spreadsheet and cross-checked for accuracy. Analysis was done using Statistical Package for Social Sciences (SPSS) version 20.0. The independent Student t-test was used to compare the mean values between COVID-19 patients and controls for parametric variables, while Mann Whitney U-test was used to compare non-parametric variables between COVID-19 patients and controls. Plasma FT3, FT4 and TSH were stratified using respective reference ranges and thyroid status determined accordingly. Chi-square test was used to assess the differences in the proportion of COVID-19 patients and controls showing thyroid dysfunction. Statistical significance was set at p < 0.05.

### Ethical consideration

A written informed consent was obtained from each participant after careful explanation of the concept and purpose of the study. Privacy and confidentiality of participants´ information were ensured. The study protocol was reviewed and approved by the FMC Abeokuta Ethics Committee (FMCA/470/HREC/01/2020/15).

## Results

### Demographic characteristics of the study population

There were no significant differences in the mean ages (p = 0.169) as well as the gender distribution (p = 0.634) between cases and controls (Table 1). The mean age of COVID-19 patients was 35.31±12.44 years, while uninfected controls had a mean age of 38.38 ± 8.08 years (Table 1). COVID-19 patients comprised of 34 (75.6%) males and 11 (24.4%) females, while the corresponding uninfected controls comprised of 32 (71.1%) males and 13 (28.9%) females.

**Table 1:** comparison of mean age and gender distribution of COVID-19 patients with uninfected healthy controls

Variable	COVID -19 patients (n=45)	Controls (n=45)	t-value	p-value
Age (years)	35.31 ± 12.44	38.38 ± 8.08	-1.387	0.169
Gender				
Male	34 (75.6)	32 (71.1)		0.634
Female	11 (24.4)	13 (28.9)		

### Plasma Concentrations of FT3, FT4, TSH and Hs-CRP

The mean plasma FT3 and TSH concentrations were significantly higher in cases compared to controls (p = 0.000; p = 0.000 respectively), whereas there was no significant difference in the mean plasma FT4 concentrations between cases and controls (p = 0.999) (Table 2). Plasma Hs-CRP concentrations were not significantly different between cases and controls (p = 0.561) (Table 2).

**Table 2:** comparison of mean plasma FT3, FT4, TSH and Hs-CRP between COVID-19 patients and uninfected healthy controls

Variable	COVID-19 patients (n=45)	Controls (n=45)	t-value	p-value
FT3 (pg/ml) Mean (SD)	4.19 (1.32)	2.42 (0.83)	7.662	0.000*
FT4 (ng/dl) Mean (SD)	1.03 (0.29)	1.03 (0.13)	0.001	0.999
TSH (μIU/ml) Mean (SD)	2.60 (1.04)	1.68 (0.67)	5.004	0.000*
Hs-CRP (mg/L) Median (IQR)	0.51(0.75)	0.42(0.39)		0.561

* Significant at p<0.05 FT3- Free tri-iodothyronine FT4- Free thyroxine TSH- Thyroid stimulating hormone Hs-CRP- High-sensitivity C-reactive protein Statistical tool: Independent t-test per FT3, FT4 and TSH (To determine if there is a statistically significant difference in mean value of each variable between cases and controls). Mann Whitney U test per Hs-CRP (Due to the non-parametric nature of this variable data, comparison was done using the non-parametric equivalent of the Independent t-test

### Thyroid status of study participants

There were significant differences in the distributions of FT3-, FT4- and thyroid- status between cases and controls (p = 0.000; p = 0.012; p = 0.004 respectively) (Table 3). Thirteen (28.9%) cases had elevated FT3 while FT3 was not elevated in any control (Table 3). Whereas 1 (2.2%) and 7 (15.6%) cases had elevated and decreased FT4 respectively, there were none with elevated or decreased FT4 among controls (Table 3). Amongst the cases were 7 (15.6) sick euthyroid and 3 (6.7%) subclinical hypothyroidism whereas none was found among the controls (Table 3).

**Table 3:** comparison of thyroid status between COVID-19 patients and apparently healthy controls

Variable	COVID-19 patients	Controls	p-value
FT3 status			
Decreased	0 (0.0)	0 (0.0)	0.000*
Normal	32 (71.1)	45 (100.0)	
Elevated	13 (28.9)	0 (0.0)	
FT4 status			
Decreased	7 (15.6)	0 (0.0)	0.012*
Normal	37 (82.2)	45 (100.0)	
Elevated	1 (2.2)	0 (0.0)	
TSH status			
Decreased	0 (0.0)	0 (0.0)	0.078
Normal	42 (93.3)	45 (100.0)	
Elevated	3 (6.7)	0 (0.0)	
Thyroid status			
Euthyroid	35 (77.8)	45 (100.0)	0.004*
Sick Euthyroid	7 (15.6)	0 (0.0)	
Subclinical hypothyroidism Hypothyroidism Hyperthyroidism	3 (6.7) 0 (0.0) 0 (0.0)	0 (0.0) 0 (0.0) 0 (0.0)	

* Significant at p<0.05 FT3- Free tri-iodothyronine FT4- Free thyroxine TSH- Thyroid stimulating hormone Statistical tool: Chi-square test (To determine if there are differences in the distribution of thyroid status between cases and controls)

## Discussion

SARS-CoV-2 infection has been reported to have detrimental effects on multiple organ systems [10, 17], including the hypothalamic-pituitary-thyroid (HPT) axis [12, 14]. Although it is suggested that the thyroid gland may be a target for SARS-CoV-2 [16], the mechanism and its effect on thyroid function are still unclear, more so in an African setting. Hence, this present study aimed at measuring plasma levels of FT3, FT4, TSH and Hs-CRP in COVID-19 patients and apparently healthy uninfected controls. The mean plasma TSH and FT3 concentrations were significantly higher in COVID-19 patients compared to controls, with sick euthyroid and subclinical hypothyroidism observed in 15.6% and 6.7% of the COVID-19 patients respectively. Higher mean plasma TSH and FT3 in COVID-19 patients compared with apparently healthy controls observed in this study could be a secondary adaptive response of the HPT function to infection with SARS-CoV-2 rather than an indication of damage to the HPT axis which should result in lower to undetectable levels of these hormones. Thyroid function abnormalities resulting from deregulation of thyroid hormone metabolism during acute and chronic illnesses have been reported to lead to changes in thyroid hormone levels that are adaptive changes rather than intrinsic abnormalities in HPT function [18-21].

Mediators of changes in thyroid function during acute and chronic illnesses are much varied, but pro-inflammatory cytokines including IL-1, IL-6 and TNF-α have been implicated in several infectious diseases [19, 22]. Increase in the levels of these cytokines has been associated with suppression of the HPT axis [23, 24]. Although the release of large amounts of pro-inflammatory cytokines described as cytokine storm syndrome, which correlates with lung injury, multi-organ failure and unfavorable prognosis of severe COVID-19 cases have been documented [25, 26], reports of pro-inflammatory cytokines levels in patients in this part of the world has been sparse. The finding of higher TSH and FT3 levels in COVID-19 patients in this present study rather than suppressed levels may be suggestive of a lesser degree or absence of the cytokine storm syndrome in these patients. Higher TSH and FT3 levels observed in this present study is in contrast with lower TSH and TT3 levels reported by Wang *et al*. [13], who also observed that the degree of decrease in TT3 correlated with disease severity. The reason for the difference in the finding of this study and that of Wang *et al*. is still unclear, but may be due to environmental or possibly genetic factors in the patient population studied. Other studies that reported significantly decreased TSH levels [13, 14], suggested that this finding in SARS patients could not be explained by the destruction of thyroid follicular epithelium since low serum levels of T3 and T4 would usually lead to increased TSH level given intact hypothalamus-pituitary function.

Muller and co-workers, reported that a substantial proportion of patients with COVID-19, requiring high intensity of care, presented with thyrotoxicosis and low serum TSH concentration, possibly as a consequence of subacute thyroiditis induced by SARS-CoV-2, in an underlying setting of non-thyroidal illness syndrome [27]. Though our finding of increased FT3 level agrees with the first case report [16] of subacute thyroiditis in an Italian asymptomatic COVID-19 patient who had high level of FT3, it is important to note that the patient presented after completion of treatment whereas in this study patients were studied before treatment commenced. The present study also showed that majority 35 (77.8%) of COVID-19 patients studied were euthyroid. This is suggestive of minimal or no damage to the thyroid gland of COVID-19 patients in the study environment. Yao *et al*. [15] in a study done in China reported that patients with SARS-CoV-2 had no remarkable changes in morphology of thyroid gland. However, another study by Wei *et al*. [12], also in China, showed that autopsies of five SARS cases revealed follicular epithelial damage in the thyroid gland with large numbers of cells exfoliated into the follicle and undergoing apoptosis, likely signalling a destructive effect of the virus on the thyroid gland. Although the clinicopathologic characteristics of the novel coronavirus is still unfolding, it has however been observed that the mortality rate is lower in Africans. Various reasons have been proposed for this observation, but it is still largely unclear and its effect on the thyroid gland is equally yet to be fully elucidated in our setting.

The present study showed that 16.5% of COVID-19 patients had sick euthyroid syndrome, while 6.7% had subclinical hypothyroidism. The ‘sick euthyroid´ syndrome observed in some of these patients may be in line with the usual finding in severely ill patients. Dworakowska and co-worker also noted that in patients severely affected by COVID-19, changes in thyroid function may relate to a ‘sick euthyroid´ syndrome, but there may be specific thyroid-related damage which requires further investigation [28]. Various mechanisms have been proposed to contribute to the development of this condition, including alterations in the iodothyronine deiodinases, TSH secretion, transport of thyroid hormone in peripheral tissues, thyroid hormone receptor activity changes and thyroid hormone binding to plasma proteins [29]. Furthermore, the syndrome may be a complex mix of physiologic adaptation and pathologic response to acute illness [29]. Subclinical hypothyroidism reported in a few of the patients in our study, may represent an early stage of thyroid dysfunction. It was likewise observed in the present study that none of the patients showed biochemical evidence of hyper or hypothyroidism. This is in contrast with a study by Leow *et al*. [14] who reported that 6.7% of SARS patients were biochemically hypothyroid; three cases with central and the remaining one with primary hypothyroidism. The strength of the present study lies in being among the first studies with efforts to determine the extent of affectation of the thyroid axis by the novel SARS-CoV-2 agent in Nigeria. Being a single-point study, the limitations include the inability to measure the levels of thyroid hormones during treatment and post recovery in order to ascertain the pattern of changes, if any. The sample size may be another limitation, hence larger multi-center studies may be needed in this area.

## Conclusion

The majority of the patients in the present study were found to be euthyroid. However, significantly higher mean plasma levels of TSH and FT3, sick euthyroid syndrome and subclinical hypothyroidism noted among some COVID-19 patients could be due to adaptive changes, intrinsic abnormalities of the thyroid gland or damage to the hypothalamic-pituitary thyroid function. Hence, there is need to pay attention to thyroid function both in the course of the infection and in the follow-up period. Again, more studies are needed to further delineate the pathophysiologic mechanisms of thyroid involvement in SARS-CoV-2 infection.

### What is known about this topic

- SARS-CoV-2 infection has been reported to have a multiple organ system affectation;
- Available reports on thyroid affectation are sparse and varied.

### What this study adds

- The COVID-19 patients studied were mostly euthyroid at diagnosis;
- Sick euthyroid syndrome and subclinical hypothyroidism were also noted among some COVID-19 patients at diagnosis.
